# Opposing Mechanisms Support the Voluntary Forgetting of Unwanted Memories

**DOI:** 10.1016/j.neuron.2012.07.025

**Published:** 2012-10-18

**Authors:** Roland G. Benoit, Michael C. Anderson

**Affiliations:** 1 MRC Cognition and Brain Sciences Unit, 15 Chaucer Road, Cambridge CB2 7EF, UK

## Abstract

Reminders of the past can trigger the recollection of events that one would rather forget. Here, using fMRI, we demonstrate two distinct neural mechanisms that foster the intentional forgetting of such unwanted memories. Both mechanisms impair long-term retention by limiting momentary awareness of the memories, yet they operate in opposite ways. One mechanism, direct suppression, disengages episodic retrieval through the systemic inhibition of hippocampal processing that originates from right dorsolateral prefrontal cortex (PFC). The opposite mechanism, thought substitution, instead engages retrieval processes to occupy the limited focus of awareness with a substitute memory. It is mediated by interactions between left caudal and midventrolateral PFC that support the selective retrieval of substitutes in the context of prepotent, unwanted memories. These findings suggest that we are not at the mercy of passive forgetting; rather, our memories can be shaped by two opposite mechanisms of mnemonic control.

## Highlights

► Voluntarily forgetting is mediated by two opposite mechanisms ► These mechanisms recruit distinct PFC systems to either engage or disengage retrieval ► DLPFC effectively inhibits retrieval processes supported by the hippocampus ► A cPFC-mid-VLPFC circuit biases retrieval to supplant unwanted memories

## Teaser

Suppressing awareness of an unwanted memory can cause forgetting. Benoit and Anderson show how this motivated forgetting can be achieved by opposite prefrontal mechanisms that either suppress hippocampal retrieval processes or engage retrieval to occupy awareness with a substitute memory.

## Introduction

The ability to remember one’s past is a two-sided coin. It allows us to relive cherished episodes but also confronts us with past events that we would rather forget. Research over the last decade indicates that this latter side is, to some degree, under voluntary control. When people confront an unwelcome reminder of a past event, they can exclude the unwanted memory from awareness. This process, in turn, impairs retention of the suppressed memory (Anderson and Green, 2001; Hertel and Calcaterra, 2005; Anderson and Huddleston, 2011). Though recent studies have started to elucidate the neural basis of this phenomenon (Anderson et al., 2004; Depue et al., 2007; Butler and James, 2010), they all leave a fundamental question unanswered: what exactly are the neurocognitive mechanisms that underlie memory suppression? The present fMRI experiment scrutinized the existence of two possible routes to forgetting unwanted memories. Both of these putative mechanisms are hypothesized to induce forgetting by limiting momentary awareness of an unwanted memory, yet they achieve this function in fundamentally opposite ways that are mediated by different neural networks.

One way to exclude a memory from awareness would be to inhibit the retrieval process directly (Bergström et al., 2009). If such *direct suppression* were possible, it may be mediated by a disruption of mnemonic processes supported by the hippocampus (HC), a structure known to be critical to conscious recollection (Squire, 1992; Eldridge et al., 2000; Eichenbaum et al., 2007). In support of this hypothesis, blood oxygen level-dependent (BOLD) signal in the HC is typically reduced during attempts to limit awareness of a memory compared with attempts to recall a memory (Anderson et al., 2004; Depue et al., 2007; Butler and James, 2010). Thus, these situations might recruit a direct suppression mechanism that disengages retrieval processes supported by the HC (cf. Anderson et al., 2004). At the same time, attempts to exclude a memory from awareness are associated with increased activation in right dorsolateral prefrontal cortex (DLPFC; approximating Brodmann area [BA] 46/9; Anderson et al., 2004; Depue et al., 2007; Butler and James, 2010), and a stronger recruitment of this region predicts greater subsequent forgetting of the avoided memories (Anderson et al., 2004; Depue et al., 2007). Importantly, across individuals, greater DLPFC activation correlates with decreased HC activation (Depue et al., 2007). This pattern suggests that the DLPFC may inhibit HC processing to prevent the retrieval of unwanted memories and that precluding awareness in this fashion impairs the suppressed memory traces (Anderson et al., 2004). However, it is unknown whether the activation changes in these two regions reflect such direct suppression attempts, and whether they indeed compose a functional network that supports retrieval inhibition. Here, using dynamic causal modeling, we examine the hypothesis that a negative DLPFC-HC coupling mediates such a mechanism of voluntary forgetting.

The opposite way of excluding an unwanted memory from awareness would be to occupy the limited focus of awareness with another competing thought, such as another memory (Hertel and Calcaterra, 2005). Because such *thought substitution* requires an alternative memory to be retrieved, it would presumably engage HC processing, not disengage it. It therefore could not be based on a systemic inhibition of this structure. Instead, this mechanism requires the selection between the substitute memory and the prepotent, unwanted memory. Previous research indicates that selective retrieval can weaken competing memory traces (Anderson et al., 1994; Norman et al., 2007) and that it is supported by two prefrontal regions (Wimber et al., 2008). One of these approximates to left BA 44/9. This part of caudal PFC (cPFC) is engaged during the retrieval of weak memories in the context of stronger, interfering memories (Wimber et al., 2008; Kuhl et al., 2008). Greater activation in cPFC has also been linked to reduced proactive interference from intruding memories in working memory tasks (Nee and Jonides, 2008). Accordingly, this region may also support processes that enable substitute recall while weakening the trace of the avoided memory. The second structure, left midventrolateral PFC (mid-VLPFC; approximating posterior parts of BA 45), has been implicated in the selection of a target from among retrieved memories (Kuhl et al., 2007, 2008; Badre and Wagner, 2007). Thus, controlling awareness of unwanted memories by thought substitution may be achieved by cooperative interactions between left cPFC and mid-VLPFC that bias retrieval toward the selective recollection of distracting substitute thoughts that occupy awareness.

**Figure 1:**
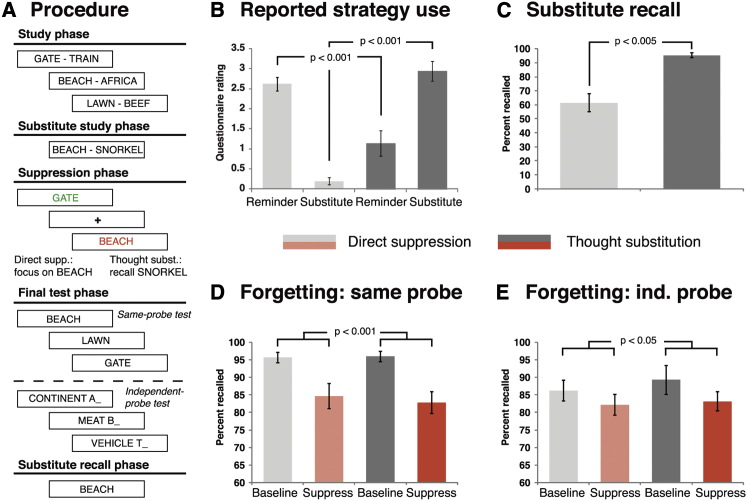
Memory Performance Indicates that Direct Suppression and Thought Substitution Induce Indistinguishable Forgetting despite Differences in People’s Reports of the Processes Engaged (A) Phases of the procedure. In the study phase, participants encoded reminder-memory word pairs. For a subset of the reminders, they then received substitute memories. During the scanned suppression phase, participants recalled some of the memories (reminders presented in green) and suppressed others (reminders presented in red). Critically, the thought substitution group did this by recalling the substitute memories, whereas the direct suppression group focused on the reminder while attempting to block out both the unwanted memory and its substitute. In a later test, they were asked to remember all words that they had previously suppressed, recalled, or had initially learned but had not seen during the suppression phase (baseline items). Finally, they also recalled all substitutes. (B and C) As expected, the groups differed in reported strategy use (i.e., focusing on the reminder as it appeared on the screen versus on the retrieved substitute while avoiding thoughts of the original memory) (B), and thought substitution yielded a greater recall rate for substitute memories, suggesting that this group practiced their retrieval (C). (D and E) The two mechanisms led to significant below-baseline forgetting on the same-probe (D) and independent-probe (E) tests. Data are represented as mean ± SEM.

To scrutinize the two putative mechanisms of voluntary forgetting, two groups of participants encoded reminder-memory pairs (e.g., BEACH-AFRICA). Participants then received substitute memories for a subset of these reminders (e.g., BEACH-SNORKEL) (Figure 1A). Afterward, they were scanned by fMRI while they recalled some of the associates and suppressed others (Anderson and Green, 2001). Critically, one group accomplished this in a manner likely to engage the hypothesized direct suppression mechanism. These participants attended to the reminder on the screen (e.g., BEACH) while trying to prevent retrieval of the associated memory (e.g., AFRICA). They were carefully instructed to not engage in any distracting activity (Bergström et al., 2009). If the memory entered awareness inadvertently, they were asked to block it out. By contrast, the other group performed a task likely to engage the thought-substitution mechanism, i.e., they recalled the substitute memory (e.g., SNORKEL) to help them preclude or supersede awareness of the to-be-avoided memory (e.g., AFRICA) (Hertel and Calcaterra, 2005). Afterward, we tested the mnemonic consequences of these mechanisms by probing retention of the suppressed, recalled, and baseline memories (i.e., items that were initially learned but not encountered during the suppression phase). We gauged the existence of these two opposing neurocognitive mechanisms first by examining whether they are supported by selective engagements of the hypothesized brain structures, and then by determining whether these structures compose functional networks that could mediate voluntary forgetting.

## Results

### Behavioral Results

#### Distinct Characteristics of Direct Suppression versus Thought Substitution

Debriefing confirmed that the thought substitution group predominantly controlled awareness of the unwanted memories by retrieving the substitutes (Figure 1B). The direct suppression group, by contrast, reported that they controlled awareness by focusing on the reminder as it appeared on the screen while attempting to inhibit the memory. The group differences were significant (substitute focus: t_(32)_ = 10.59, p < 0.001; reminder focus: t_(32)_ = −4.12, p < 0.001), suggesting that participants performed the tasks as instructed.

These self-reports were also corroborated by an objective measure, i.e., recall of the substitute memories after the suppression and final test phases (Figure 1C). It has been shown that repeated retrieval benefits retention (Roediger and Butler, 2011), and indeed the thought substitution group recalled nearly all the substitutes. In comparison, the direct suppression group remembered far fewer substitutes (t_(34)_ = 5.63, p < 0.005). This pattern is consistent with the expectation that only the thought substitution group practiced retrieving those memories.

#### Direct Suppression and Thought Substitution Cause Below-Baseline Forgetting

To assess the mnemonic consequences of direct suppression and thought substitution, we asked participants to remember all *suppress* and *recall* words at the end. Moreover, they recalled *baseline* items, which they had initially encoded but which were not cued during the suppression phase. The recall rate for these items constitutes a baseline of forgetting due to the passage of time that occurs without any suppression attempts. Both mechanisms led to significant forgetting below this baseline when memory was probed with the original reminder (*same-probe* [SP] test; e.g., cue with BEACH for AFRICA; Figure 1A). This was revealed by a two-way ANOVA with the within-subject factor retrieval status (baseline, recall, suppress) and the between-subject factor group (thought substitution, direct suppression), which yielded a significant effect of retrieval status only (F_(2,,68)_ = 21.5, p < 0.001). This effect partly reflected below-baseline forgetting of the suppressed memories, as shown by a follow-up ANOVA comparing recall for baseline versus suppress items (F_(1,34)_ = 23.1, p < 0.001). This effect also did not interact with group (F_(1,34)_ < 1). (For further analyses, see Supplemental Information available online.)

Although the same-probe test results suggest that the suppressed memories (e.g., AFRICA) were inhibited, they could also reflect the action of other mechanisms, such as unlearning of the reminder-memory associations (Anderson, 2003). In a second test, we therefore cued the memories with pre-experimentally existing probes, i.e., the memories’ categories plus their first letter (e.g., CONTINENT-A for AFRICA). A similar result emerged on this *independent-probe* (IP) test (Figure 1E). The initial ANOVA with all three conditions revealed a trend for a main effect (F_(2,68)_ = 2.59, p < 0.09), and the critical ANOVA limited to baseline and suppress items confirmed significant below-baseline forgetting (F_(1,34)_ = 4.24, p < 0.05). Again, this effect did not vary by group (F_(1,34)_ < 1). The generalization of forgetting to this independent-probe test indicates a disruption of the trace itself rather than merely a weakening of particular associations into it (Anderson, 2003). Thus, two mechanisms for suppressing awareness of unwanted memories that are phenomenologically completely different caused behaviorally indistinguishable forgetting. Next, we examined whether memory control in the two groups was supported by the same neural network, or whether it was mediated instead by the hypothesized dissociable neural mechanisms.

### Neuroimaging Results

#### Distinct Regions Contribute to Direct Suppression versus Thought Substitution

To examine whether the two groups exhibited selective activation patterns consistent with the hypothesized mechanisms, we report average contrast estimates from a priori regions of interest (ROIs; see Experimental Procedures; Tables S1–S4 for exploratory whole-brain analyses). Thereby, the analyses are not biased in favor of any group (Kriegeskorte et al., 2009). For the directed between-group predictions, we performed one-tailed tests as indicated below. We first concentrate on right DLPFC and HC, the brain areas hypothesized to mediate direct suppression, before turning to left cPFC and mid-VLPFC, the regions hypothesized to be involved in thought substitution.

#### Direct Suppression Is Associated with Right DLPFC Recruitment and HC Disengagement

**Figure 2:**
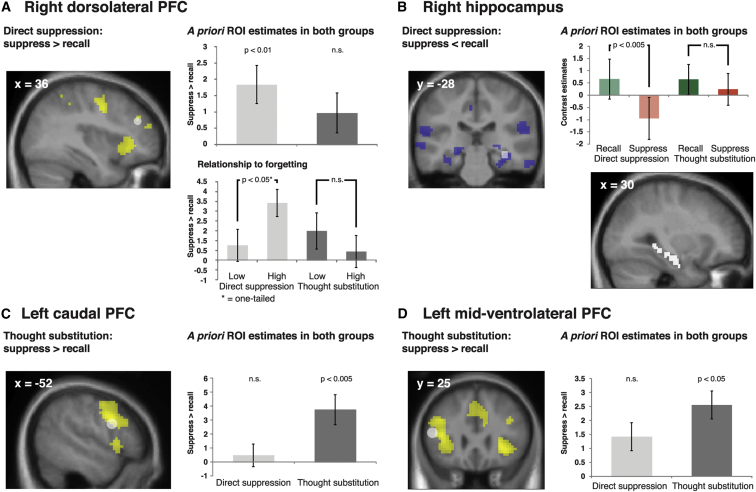
Region-of-Interest Analyses Indicate that Direct Suppression and Thought Substitution Engage Distinct Prefrontal Regions and that Only Direct Suppression Leads to Reduced Hippocampal Activation Regions contributing to direct suppression versus thought substitution, as revealed by contrast estimates from the respective a priori regions of interest (ROIs). (Accompanying whole-brain maps at the left side of each panel are provided for illustrative purposes, thresholded at p < 0.001, uncorrected, and at least five contiguous voxels. The ROIs are marked in white.) (A) The task likely to engage direct suppression was associated with increased activation in right dorsolateral PFC. Moreover, for this group only, activation was greater for individuals who forgot more of the suppressed memories (i.e., greater BOLD signal changes for high versus low forgetters). (B) Only the direct suppression group also exhibited decreased activation in right hippocampus during suppress versus recall events. (C and D) By contrast, only the thought substitution group showed increased activation in both left caudal PFC (C) and left midventrolateral PFC (D). Data are represented as mean ± SEM; n.s., not significant; see also Figure S1 and Tables S1–S4.

First, attempts to suppress retrieval directly were associated with greater right DLPFC activation than were recall attempts (Figure 2A; t_(17)_ = 3.14, p < 0.01). Moreover, consistent with previous results (Anderson et al., 2004), engagement of this DLPFC region was stronger for individuals who successfully induced more below-baseline forgetting of unwanted memories. This was confirmed by a significant median split based on memory inhibition scores (Figure 2A; t_(16)_ = −2, p < 0.05, one-tailed). By contrast, the thought substitution group exhibited neither greater DLPFC activation for suppress versus recall events (Figure 2A; t_(17)_ = 1.59, p = 0.131) nor a modulation of this effect by forgetting (Figure 2A; t_(16)_ = 0.85, p = 0.203, one-tailed; if anything, there was greater activation for the low forgetters). Consequently, the relationship between DLPFC recruitment and forgetting trended to be stronger for the direct suppression group than it was for the thought substitution group (interaction group × forgetting: F_(1,32)_ = 3.85, p = 0.058). These findings are consistent with a greater involvement of DLPFC in direct suppression than in thought substitution. It should be noted, however, that exploratory brain analysis (with an uncorrected threshold of p < 0.001 and at least five contiguous voxels) also revealed an effect for the thought substitution group in a more caudal DLPFC region, although this effect did not survive whole-brain or small-volume FWE correction (in contrast to the effect for the direct suppression group, which remained significant; Tables S1–S4).

Second, the right hippocampal ROI also showed the expected effects. Activation in the HC was decreased during suppress compared with recall events for the direct suppression (Figure 2B; t_(17)_ = 3.53, p < 0.005) but not for the thought substitution group (Figure 2B; t_(17)_ = 0.81, p = 0.429). Moreover, the activation difference for the suppress versus recall conditions indeed differed between the two groups (t_(34)_ = −1.78, p < 0.05, one-tailed). (A similar significant effect emerged for the left hippocampus; Supplemental Information and Figure S1.) Thus, only the task likely to engage the direct suppression mechanism was associated with increased DLPFC and decreased HC activation. These findings support the hypothesis that attempts to prevent retrieval are supported by a neural circuit that achieves retrieval inhibition.

#### Thought Substitution Is Associated with Left cPFC and Mid-VLPFC Recruitment

By contrast, attempts to suppress awareness of an unwanted memory through thought substitution were associated with significant engagement of the two hypothesized left prefrontal regions. The thought substitution group exhibited greater cPFC activation for suppress than recall events (Figure 2C; t_(17)_ = 3.48, p < 0.005). This effect was not present during direct suppression (Figure 2C; t_(17)_ = 0.59, p = 0.566), and the group difference was significant (t_(34)_ = −2.43, p < 0.05, one-tailed). As predicted, a similar pattern emerged for the mid-VLPFC ROI, with an effect of suppress versus recall for the thought substitution (Figure 2D; t_(17)_ = 2.78, p < 0.05) but not the direct suppression group (Figure 2D; t_(17)_ = 1.38, p = 0.185), though the group difference was not significant (t_(34)_ = 0.82, p = 0.21, one-tailed).

Thus, the two memory suppression tasks were indeed associated with BOLD signal changes in those brain structures hypothesized to support the two opposite mechanisms of voluntary memory control. Moreover, the involvement of most areas differed between the groups. This was corroborated by an ANOVA on the contrast estimates for suppress versus recall events with the factor ROI (DLPFC, HC, cPFC, mid-VLPFC) and group (thought substitution, direct suppression) that yielded the significant interaction (F_(3,102)_ = 7.79, p < 0.05).

#### DLPFC Exerts Inhibitory Control on the Hippocampus during Direct Suppression

The direct suppression group exhibited stronger DLPFC engagement and reduced HC activation during suppression. This finding is consistent with the hypothesized mechanism of retrieval inhibition, in which the former region exerts inhibitory control over processes supported by the latter. To formally test for a negative influence of DLPFC on HC activation, we scrutinized the interactions between these regions with dynamic causal modeling (Friston et al., 2003). First, we investigated whether the data can best be accounted for by models that include the hypothesized “top-down” influence during suppression; we then examined the nature of this putative inhibitory connection and its relationship to subsequent forgetting of suppressed memories. (Note that it was not possible to apply dynamic causal modeling to the thought substitution data, because, as predicted, this group did not exhibit any significant suppress versus recall effects on HC and DLPFC BOLD signal [Stephan et al., 2010].)

**Figure 3:**
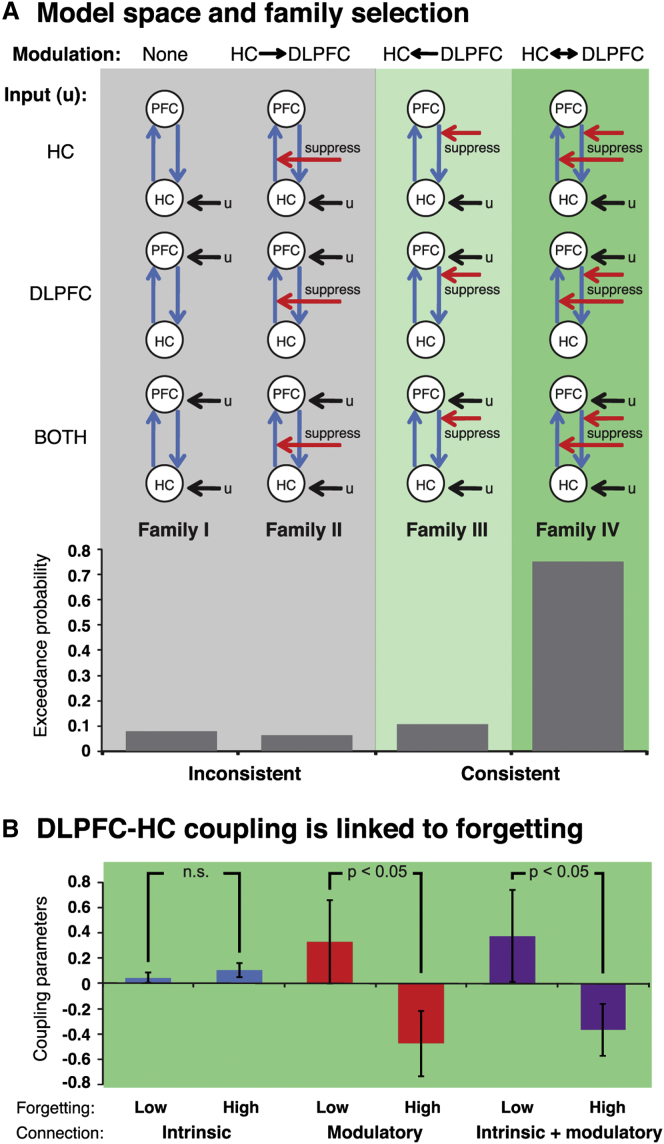
Effective Connectivity Analyses Establish a Modulatory Influence of DLPFC on the Hippocampus during Direct Suppression that Is Stronger when People Are Better at Suppressing Memories Dynamic causal modeling of the relationship between right dorsolateral prefrontal cortex (DLPFC) and hippocampus (HC) during direct suppression. (A) Each family comprised three models that varied in the location of the driving input (i.e., either via the HC, DLPFC, or both nodes), and the families differed in the connections that could be modulated during suppress events. Families I and II (gray background) did not include a modulatory component from the DLPFC to the HC, whereas families III and IV (green background) did comprise such a “top-down” modulatory connection. Thus, only the latter two families are consistent with the hypothesized increased DLPFC influence on HC activation during direct suppression, and indeed random-effects Bayesian model selection indicated that family IV could account best for the data. (B) Coupling parameters of the connection from DLPFC to HC, derived from Bayesian model averaging of the winning family IV. The modulation of the connectivity as well as the absolute effective connectivity during suppression (i.e., the sum of the modulatory plus intrinsic component) differed for those participants who forgot more versus less suppressed memories. Data are represented as mean ± SEM; see also Figure S2.

We composed a basic network consisting of the two nodes, bidirectional intrinsic connections and inhibitory autoconnections. Any reminder onsets could elicit responses in the network. The exact location of this driving input was varied across three model types, i.e., it entered the network via the HC, the DLPFC, or both nodes. We then constructed four model families, each of which contained all three model types. Importantly, the families varied in the connection that could be modulated by memory suppression (Figure 3A). Family I did not have any such modulatory component, family II included a modulation of the “bottom-up” connection from HC to DLPFC, family III exhibited the reverse, “top-down” modulatory component (i.e., from DLPFC to HC), and family IV allowed both connections to be modulated by suppress events. Critically, only the latter two families are consistent with the putative inhibitory mechanism. (Note that modeling DLPFC-HC interactions does not presuppose that these regions exhibit monosynaptic connections. Rather, the resulting coupling parameters represent their effective connectivity, which may well be mediated by relay nodes [Stephan et al., 2010; Friston, 1994]. However, including such nodes, e.g., the retrosplenial cortex, may potentially change aspects of the estimated connectivity pattern.)

On the estimated models, we ran Bayesian model selection (BMS) in a random-effects approach to identify the family most likely to have generated the data (Penny et al., 2010). (Note that BMS penalizes for the degree of model complexity.) The analysis indicated that family IV could account best for the data, with an exceedance probability (EP) of 0.75 (Figure 3A). (A fixed-effects analysis provided very strong evidence for the same family. Moreover, this family was also selected when the model space was first partitioned into two metafamilies that were either consistent [III and IV] or inconsistent [I and II] with the hypothesized “top-down” modulation [Supplemental Information and Figure S2].) Thus, the winning family shares a structure consistent with the hypothesized increased influence of DLPFC on HC activation during direct suppression. However, a follow-up BMS, based on the three members of family IV, was unable to determine a superior model within that family (EP: input via HC: 0.51; DLPFC: 0.39; both nodes: 0.1), suggesting that the exact location of the driving input had little impact on the model evidence.

The proposed mechanism further posits that DLPFC exerts a negative influence on HC engagement. The resulting reduction in hippocampal processing, in turn, would then induce forgetting of the suppressed memory items that exceeds the forgetting arising as a passage of time. Thus, the “top-down” connectivity from DLPFC to HC during suppress events should be negative especially for individuals who forget more of the suppressed memories (relative to the baseline memories). To test this account, we performed Bayesian model averaging (BMA) on the winning family IV (Penny et al., 2010). This procedure computes weighted averages of each model parameter, in which the weighting is determined by the posterior probability of each model. We then conducted three analyses. The first examined the intrinsic connectivity from DLPFC to HC, i.e., the coupling that is not modulated by suppress events. These parameters should not necessarily be related to suppression success, and indeed they did not differ between participants who forgot more or less suppressed memories (median split: t_(16)_ = −0.91, p = 0.378) (Figure 3B). By contrast, the parameters indicating the change in coupling during suppression should differ according to the degree of below-baseline forgetting. That is, individuals who forget more unwanted memories should show evidence of greater inhibitory (i.e., negative) modulation by DLPFC on HC. This was observed in the present data, in which the modulatory coupling parameters differed for high and low forgetters (t_(16)_ = 1.92, p < 0.05, one-tailed) (Figure 3B), and indeed they yielded a strong trend to be negative for the high forgetters (t_(8)_ = −1.84, p < 0.052, one-tailed). In contrast, the parameters were not reliably positive or negative for the low forgetters (t_(8)_ = 1, p = 0.346).

The same pattern emerged for the absolute connectivity from DLPFC to HC during direct suppression, i.e., the sum of the intrinsic and modulatory connections (Figure 3B). Again, parameters for the high and low forgetters differed significantly (t_(16)_ = 1.77, p < 0.05, one-tailed), and they showed a trend for a negative influence of DLPFC on HC activation in the high forgetters only (high forgetters: t_(8)_ = −1.77, p = 0.057, one-tailed; low forgetters: t_(8)_ = 1.03, p = 0.334).

Thus, our data indicate that those models can account best for the direct suppression data that are consistent with the proposed retrieval inhibition mechanism. That is, they entail a modulation of the connection from DLPFC to HC during memory suppression. Moreover, the coupling parameters showed the expected relationship with forgetting. Critically, individuals who forgot more of the suppressed memories also exhibited a stronger effective connectivity between the two regions. These connections showed a strong trend to be negative, i.e., according to dynamic causal modeling increased DLPFC recruitment caused reduced hippocampal activation.

#### cPFC-Mid-VLPFC Coupling Is Linked to the Resolution of Memory Competition during Thought Substitution

As predicted, suppressing awareness of unwanted memories via thought substitution led to increased left cPFC and mid-VLPFC activation. We further hypothesized that these regions would interact to resolve competition in favor of the thought substitute over the avoided memory. If increased cPFC-mid-VLPFC coupling supports such a mechanism, it should be stronger (1) for individuals who found it more difficult to substitute the competing, unwanted memories with the alternative memories and (2) for those who had to continue engaging this mechanism throughout the whole experiment because they forgot less of the competing, unwanted memories.

**Figure 4:**
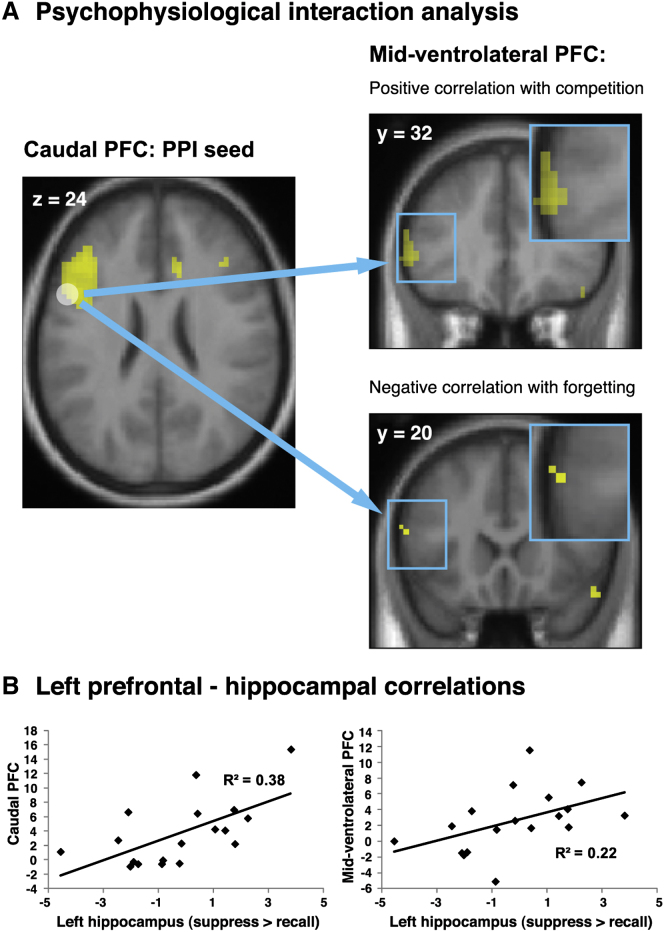
Connectivity Analyses Reveal the Importance of a Left Prefrontal Circuit in Thought Substitution and Suggest that Recruitment of These Regions Is Positively Related to Hippocampal Activation (A) Psychophysiological interaction between left caudal PFC (seed) and midventrolateral PFC during thought substitution. Coupling between these regions was stronger in case of greater competition between the avoided versus substitute memory (top) and weaker in case of greater forgetting, i.e., when the forgotten memory item did not interfere with the retrieval of its substitute (bottom). For illustration, the SPMs are thresholded at p < 0.005, uncorrected, and at least five contiguous voxels. The effects are significant after FWE correction for midventrolateral PFC. (B) Contrast estimates for suppress versus recall events correlated between the left hippocampus and both caudal and midventrolateral PFC, suggesting a functional link between retrieval processes supported by the hippocampus and retrieval control processes supported by the two prefrontal regions.

Because we did not have any strong prediction regarding the causal directionality of the coupling, we employed a psychophysiological interaction (PPI) approach that does not require such assumptions (Friston et al., 1997). We first performed a PPI analysis to reveal those regions showing greater functional coupling with left cPFC during suppress than recall events and then conducted regression analyses of the coupling parameters within mid-VLPFC to test the two predictions (Benoit et al., 2011). First, we examined whether the regions are indeed more strongly coupled in cases when participants reported greater difficulty in using the substitutes to control awareness of the unwanted memory, as these situations require a greater engagement of a system that resolves memory competition. Therefore, for each participant, we computed the ratio of (1) the difficulty to remember the substitutes versus (2) the ease to suppress the original memories (as indexed on the postexperiment questionnaire; see Experimental Procedures). This procedure yields greater scores for those who found it more difficult to remember the substitutes and simultaneously suppress the unwanted memories. Consistent with our prediction, the analysis revealed a positive correlation between this competition score and coupling parameters within mid-VLPFC (Figure 4A; X, Y, Z: −57, 32, 13; z = 3.4; FWE small-volume corrected). Thus, the two left prefrontal regions exhibited a greater increase in functional connectivity during thought substitution for individuals who found it more difficult to occupy awareness with the substitute instead of the unwanted memory.

Second, it recently has been demonstrated that regions including VLPFC are recruited less when the demands on competition resolution are reduced through prior acts of control (Kuhl et al., 2007; Wimber et al., 2011). If interactions between cPFC and mid-VLPFC contribute to overcoming the competition between the avoided memory and its substitute, one may accordingly expect a weaker coupling for individuals who successfully induced greater forgetting of unwanted memories. For these participants, there is less demand to continue engaging competition resolution, because the forgotten memories no longer interfere with substitute recall. In line with this prediction, we observed a negative correlation between below-baseline forgetting on the final test and coupling parameters in parts of mid-VLPFC (Figure 4A; −57, 20, 16; z = 3.17; FWE small-volume corrected): the more effectively people forgot unwanted memories, the less coupled mid-VLPFC was with cPFC. By contrast, there was no such relationship for the direct suppression group. Taken together, these data indicate that when people attempt to control unwanted memories by occupying awareness with a thought substitute, this mechanism is mediated by interactions between two left prefrontal regions involved in controlled memory retrieval and selection.

Moreover, if thought substitution engages processes supported by cPFC and mid-VLPFC to resolve retrieval competition, the activation in these two regions may scale with hippocampal activation. It has been argued that when one has to select between conflicting memories, hippocampal BOLD signal may reflect the concurrent activation of both relevant and irrelevant memory traces (Kuhl et al., 2007; Wimber et al., 2009), and activation in the left HC shows increased activation during the retrieval of two unrelated associations (Ford et al., 2010). By this account, greater HC activation during thought substitution would indicate that both memory traces have been activated, thus marking a greater requirement for controlled retrieval and selection of the substitute over the unwanted memory. In line with this prediction, contrast estimates for suppress versus recall events correlated between the left HC and both cPFC (r_(18)_ = 0.62, p < 0.01; Figure 4B) and mid-VLPFC (r_(18)_ = 0.47, p < 0.05; Figure 4B). Thus, individuals who exhibited greater HC activation during substitution attempts also exhibited greater cPFC and mid-VLPFC recruitment. This pattern suggests that the retrieval selection processes supported by the left-prefrontal circuit are functionally linked to retrieval processes supported by the hippocampus. By contrast, for the direct suppression group, neither cPFC nor mid-VLPFC activation correlated with left HC engagement (cPFC: r_(18)_ = 0.19, p = 0.44; mid-VLPFC: r_(18)_ = 0.06, p = 0.822). Thus, efforts to ensure that awareness is exclusively occupied by alternate thoughts are accompanied by increased activation in the hippocampus, the opposite of what occurs during the direct suppression of unwanted memories.

## Discussion

This study scrutinized two mechanisms that may underlie voluntary forgetting, i.e., direct suppression and thought substitution. Both of these are hypothesized to impair long-term retention by reducing momentary awareness of the unwanted memory, yet to accomplish this in fundamentally opposite ways: direct suppression by inhibiting the retrieval process directly and thought substitution by recruiting the retrieval process to access a distracting memory to occupy the limited focus of awareness. We employed two suppression tasks designed to engage those hypothesized mechanisms. Though the tasks were phenomenologically completely different, they both impaired later retention of suppressed memories below the recall rate for baseline items. This forgetting effect was not only observed when memory was probed with the original reminder associated with it, but also when it was cued with an alternate association, i.e., the item’s respective category plus its first letter. Thus, the forgetting cannot simply reflect unlearning of the association between the reminder and the memory and is also unlikely to result from interference from the association between the reminder and the substitute. Instead, the observed cue-independent forgetting indicates that both direct suppression and thought substitution indeed weaken suppressed memory traces (Anderson, 2003).

Though the two groups exhibited identical forgetting patterns, the neuroimaging data indicate that these memory impairments were nevertheless mediated by dissociable neural mechanisms. The direct suppression group revealed the functional network that we had hypothesized to support retrieval suppression. Specifically, effective connectivity analyses indicated that right DLPFC exerts a negative influence on hippocampal activation during suppression attempts. This modulatory influence is likely to be achieved via relays such as other medial temporal lobe structures or the retrosplenial cortex (Goldman-Rakic et al., 1984; Morris et al., 1999), given the lack of evidence for monosynaptic connections between the two regions. Neurons in the DLPFC may code for a cognitive set, i.e., direct suppression, that is implemented when a cue to suppress appears. Alternatively, implementation of the set may be triggered by the detection that, in a suppression context, a reminder starts to elicit its associated memory (a process coined “ecphory”; Tulving, 1972). Thus, the latter interpretation implies that suppression processes supported by the DLPFC are only engaged once an unwanted memory intrudes into awareness. Indeed, the model family that did account best for the data also featured a modulation of the connection from HC to DLPFC. If activation in the HC signals the retrieval of an (unwanted) memory, this information may be transferred to the DLPFC.

Moreover, both DLPFC activation and its influence on HC activation were stronger in individuals who successfully forgot more of the suppressed memories. Given the hypothesized role of the HC in recollection (Squire, 1992; Eldridge et al., 2000; Eichenbaum et al., 2007), the data thus suggest that DLPFC inhibits retrieval processes supported by that region. If so, then this inhibitory modulation might compromise the consolidation of the suppressed memory by, for example, disrupting the replay of its hippocampal representation (Karlsson and Frank, 2009; Carr et al., 2011). As a corollary, inhibition would cause forgetting of the suppressed memory, and individuals who are more effective at inhibiting retrieval would exhibit a greater degree of forgetting.

The direct suppression mechanism shown here may elucidate the causes of mnemonic disorders such as psychogenic amnesia (Tramoni et al., 2009; Kikuchi et al., 2010) but also may help to understand how people cope with intrusive memories in the aftermath of traumatic events (Shin et al., 1999; Lyoo et al., 2011). On one hand, Kikuchi et al. (2010) scanned two neurologically normal patients who could remember new experiences despite exhibiting dense psychogenic retrograde amnesia. When these patients viewed photographs of faces of acquaintances drawn from the period for which they were amnesic (faces that they did not recognize), Kikuchi et al. observed greater DLPFC and ventrolateral PFC activation as well as reduced hippocampal activation. This pattern emerged even in comparison with activation for novel faces. Thus, a hyperactivity of the DLPFC-hippocampal circuit observed here might contribute to severe memory disruptions.

On the other hand, inhibitory processes supported by DLPFC may help in coping with traumatic experiences. A recent longitudinal study examined the structural brain changes in survivors of a subway disaster, and the relation of those changes with the recovery from posttraumatic stress disorder (PTSD) (Lyoo et al., 2011). Survivors who exhibited the greatest DLPFC cortical thickness 1 year after the disaster also showed the largest reductions in PTSD symptoms. Moreover, over the course of 3 years, DLPFC volume normalized to the level of controls with the degree of recovery. Thus, processes supported by this region may foster the control of negative emotions (Ochsner and Gross, 2005) but may also be involved in coping with intrusive memories. Consistent with this idea, PTSD patients exhibit reduced DLPFC recruitment when presented with reminders of traumatic experiences (Shin et al., 1999), and our results show that less DLPFC activation can be linked to less forgetting of reminded memories (see also Anderson et al., 2004; Depue et al., 2007).

In contrast, for the thought substitution group, HC activation did not differ reliably between the suppress and recall conditions, and this reduced modulation differed from the modulation observed for the direct suppression group. Given that recalling a memory (whether the original or a substitute) probably always requires engagement of the hippocampus, this dissociation further supports the proposal that the selective HC disengagement during direct suppression reflects a systemic disruption of retrieval. Moreover, the observed pattern mirrors recent evidence from event-related potentials, showing that only direct suppression but not thought substitution attenuates the parietal positivity between 300 and 600 ms (Bergström et al., 2009; Hanslmayr et al., 2009), i.e., a component linked to successful recollection (Mecklinger, 2000). The current data suggest that this selective attenuation during direct suppression may reflect inhibited hippocampal processing.

On the other hand, precluding awareness of unwanted memories by recalling substitute memories was associated with increased activation in left cPFC and mid-VLPFC. Thus, this task recruited those regions that we hypothesized to support a mechanism of thought substitution. The two areas have respectively been implicated in the retrieval of weak memories in the context of interfering, stronger memories (Wimber et al., 2008) and in the postretrieval selection between active memory representations (Kuhl et al., 2008; Badre and Wagner, 2007). Our data indicate that when thought substitution is challenging due to increased interference from unwanted memories, the functional connectivity of these regions is greater. We observed a stronger coupling for individuals who found it more difficult to recall the alternative memory while keeping the avoided memory out of mind. This increased coupling may reflect a greater demand on control processes necessary to retrieve and select the substitute in the presence of an involuntarily recalled memory. Conversely, the connectivity was weaker for individuals who successfully forgot more of the suppressed memories. Thus, these regions are more tightly coupled in case of greater experienced competition, but less coupled in case of greater forgetting, i.e., in situations when the avoided memories do not interfere with the substitutes. This pattern is consistent with our hypothesis that precluding awareness of unwanted memories by substitution engages a mechanism of competition resolution mediated by left cPFC-mid-VLPFC interactions. Moreover, these regions were more strongly engaged in individuals that also showed greater hippocampal activation during substitution attempts. If, in this context, greater HC activation can indeed be taken to reflect the concurrent retrieval of the two competing memory traces (Kuhl et al., 2007; Wimber et al., 2009), this suggests a functional link between retrieval processes supported by the HC and retrieval selection processes mediated by cPFC and mid-VLPFC.

During thought substitution, competition from an unwanted memory may be attenuated by a direct and selective weakening of the interfering memory, which, in turn, would render it inaccessible during later retrieval attempts (Storm and Nestojko, 2010). Alternatively, competition may be attenuated by selectively strengthening the substitute thought, making it easier to access and occupy awareness. In this case, forgetting of the unwanted memory may occur via an indirect process akin to lateral inhibition in visual attention (Desimone and Duncan, 1995): the successfully activated substitute trace may weaken the competing, unwanted memory (Levy and Anderson, 2002; Norman et al., 2007). The correlations between activation in the two left prefrontal regions and the HC may suggest that these effects take place at the level of the hippocampal memory traces.

Critically, either of these accounts predicts that the effectiveness of thought substitution as an approach to forgetting depends on the relatedness of the substitute to the unwanted memory. That is, if the two memories are coded by overlapping neuronal populations, it would not be possible to completely weaken the avoided memory while strengthening the substitute trace (Norman et al., 2007; Goodmon and Anderson, 2011). In such cases, it might be more effective to engage a more systemic direct suppression mechanism. In line with this proposal, direct suppression can sometimes induce cue-independent forgetting in situations in which thought substitution fails to do so (Bergström et al., 2009). An important avenue for future research is to characterize the conditions determining the efficacy of the two mechanisms.

To conclude, there seem to be at least two routes that can lead to voluntary forgetting: a direct suppression mechanism that systemically disrupts retrieval processes and a thought substitution mechanism that impairs retention by resolving competition at the level of conflicting, individual memories. Both of these mechanisms limit momentary awareness of unwanted memories—one by suppressing representations needed to achieve awareness of a memory and the other by activating representations that occupy the limited capacity of awareness. Both ways of controlling awareness also induced, in the present study, behaviorally indistinguishable forgetting. Strikingly, despite these functional similarities, the data reported here indicate that these mechanisms are mediated by distinct neural networks that achieve their functions in very different ways. Whereas direct suppression appears to reflect hippocampal suppression originating from the DLPFC, thought substitution seems to reflect the resolution of competition mediated by cPFC-mid-VLPFC coupling and possible interactions with hippocampal retrieval processes. Appreciation of these distinct systems underlying the control of unwanted memories may help in the development of treatments that remediate mental health problems associated with a deficient regulation of memories, such as might occur in the aftermath of trauma (Dunn et al., 2009; Brewin, 2011).

## Experimental Procedures

### Participants

Forty right-handed volunteers participated. They all reported no history of psychiatric or neurological disorder and gave written informed consent as approved by the local research ethics committee. Four participants were excluded either due to excessive movement (two) or falling asleep in the scanner (two). Thus, data from 36 participants are reported, with half performing thought substitution (six males; mean age: 23.8, range: 19–31 years) and the other half direct suppression (six males; mean age: 23.7, range: 20–30 years).

### Tasks

We used a modified Think/NoThink procedure (Anderson and Green, 2001) with four phases (Figure 1A): (1) a study phase, during which participants encoded reminder-memory pairs; (2) a practice phase, during which all participants practiced both direct suppression and thought substitution on filler pairs; (3) the critical suppression phase, during which they were scanned; and (4) the final test phase, during which we tested their memory.

In the study phase, participants encoded 36 critical reminder-memory word pairs (e.g., BEACH-AFRICA). A third of those constituted the suppress items, another third the recall items, and the final third served as baseline items for the final test. Assignment of words to the three conditions was counterbalanced across participants. In addition, they also memorized a further 18 filler pairs that were used for practice. The study phase had three stages. First, each pair appeared for 3.4 s (interstimulus interval [ISI]: 600 ms). Second, participants overtly recalled the memories in response to the reminders, which were shown for up to 6 s or until a response was given. After reminder offset (and a 600 ms ISI), the correct memory appeared for 1 s. This procedure was repeated until participants recalled at least 50% of the critical memories (all succeeded within the maximum of three iterations). Third, we presented each reminder one more time for up to 3.3 s (ISI: 1.1 s), and without feedback, to assess which memories had been learned.

During practice, all participants were first trained on the task likely to engage direct suppression (Bergström et al., 2009). They were instructed to covertly recall memories for reminders presented in green font (recall condition) but to avoid thinking of memories for reminders presented in red (suppress condition). On each trial, they were required to first read and comprehend the reminder. In the recall condition, they then had to retrieve the associated memory as quickly as possible and keep it in mind while the reminder remained onscreen. By contrast, in the suppress condition, they had to block out all thoughts of the associated memory without engaging in any distracting activity. Whenever a memory intruded into awareness, they were asked to “push it out of mind.” Participants practiced the task with timings identical to the suppression phase proper. That is, suppress and recall trials alternated pseudorandomly. Each reminder was onscreen for 3 s, and the ISI was jittered (≥0.5 s; mean ± SD: 2.3 ± 1.7) to optimize the efficiency of the event-related fMRI design (as determined by optseq2, http://surfer.nmr.mgh.harvard.edu/optseq). During the ISI, a fixation cross appeared.

Afterward, all participants were trained on the task designed to engage thought substitution. For each item of the suppress condition, they encoded a substitute word that was presented with the respective reminder (e.g., BEACH-SNORKEL) for 2 s (ISI: 600 ms). (Memory for each substitute was refreshed just before and halfway through the suppression phase for 2 s.) They then practiced the task, with the instruction to avoid thinking of the memories associated with red reminders by thinking of the provided substitutes instead (Hertel and Calcaterra, 2005). If a memory intruded into awareness, they were asked to “push it out” by focusing on the substitute. During the final practice stage, half of the participants continued with thought substitution, whereas the other half were asked to engage in direct suppression and to not use thought substitution at all. This latter group had to avoid thinking of both the original memories and their substitutes. Thus, both groups received training on both putative suppression mechanisms, and they were matched in their exposure to the substitutes. Finally, participants practiced the prescribed task, and we confirmed that they performed it as instructed.

During the suppression phase, participants were scanned by fMRI for six runs. In each run, they saw each reminder of the recall and suppress condition twice, with the constraint that any reminder of a condition could only be repeated once all the others had been presented. Thus, they suppressed or recalled each memory 12 times.

In the final test phase, participants had to remember all memories, i.e., irrespective of retrieval status (suppress, recall, and baseline). The reminders were presented for a maximum of 3.3 s or until a response was given (ISI: 1.1 s). A response was coded as correct if participants recalled the memory while the cue was onscreen. In a same-probe test, memory was probed with the original reminders. A second, independent-probe test was used to test whether forgetting generalized to novel cues (Anderson 2003). Here, we cued with the semantic category of the memory and its first letter (e.g., CONTINENT-A for AFRICA). The order of these two tests was counterbalanced. Finally, we tested memory for the substitutes with an SP procedure.

During debriefing, participants rated on a 5 point scale for each suppress item the degree to which they had focused (1) on the reminder as it appeared on the screen and (2) on the substitute (0: never; 4: all the time). (These data were only collected for 34 of the 36 participants.) For each item, they also indicated on a 5 point scale their difficulty in (1) suppressing the memory and (2) suppressing or recalling, respectively, the substitute word (1: not difficult at all; 5: very difficult).

### Behavioral Analyses

Final recall for suppress, recall, and baseline items was analyzed relative to the number of successfully learned words. Thus, analyses indicate the percentage of words remembered conditional on correct initial learning. To examine relationships between forgetting and brain activation, we normalized below-baseline forgetting (expressed as recall performance for baseline minus suppress items) within each of the three counterbalancing conditions. This was done separately for the SP and IP data. We then averaged the forgetting scores of the two tests to get our index of forgetting.

### fMRI Data Acquisition and Preprocessing

A 3T Siemens TIM Trio MRI scanner was used for acquisition of T2^∗^-weighted echoplanar images (64 × 64; 3 × 3 mm pixels; 3 mm thick, oriented to the AC-PC plane; TR: 2 s; TE: 30 ms; flip angle 78°; 133 volumes for each of the six sessions). Additionally, MPRAGE structural images were acquired (256 × 240 × 192; 1 mm^3^ isotropic voxels; TR: 2,250 ms; TE: 2.99 ms; flip angle 9°).

Data were analyzed using SPM8 (http://www.fil.ion.ucl.ac.uk/spm/software/spm8). The volumes were realigned, corrected for different slice acquisition times, and coregistered with the structural images. These were spatially normalized and the resulting parameters served to normalize the functional images into 3 × 3 × 3 mm^3^ cubic voxels by fourth degree B-spine interpolation (using the Montreal Neurological Institute reference brain). The images were then smoothed by an isotropic 8 mm FWHM Gaussian kernel.

### fMRI Analyses

#### Regional Activation

The variance in BOLD signal was decomposed in a general linear model (Friston et al., 1995), separately for each run. Delta functions coded the time point of reminder onsets, separately for suppress and recall events. These regressors included only those reminders whose associates had successfully been learned. Reminders for the remaining items were coded by two additional regressors (one for each condition). A further delta function coded transient changes associated with block onset. All of those regressors were convolved with the canonical hemodynamic response function. The full model additionally comprised regressors representing the mean over scans and residual movement artifacts. A 1/128 Hz high-pass filter was applied to the data and the model. Parameters for each regressor were estimated from the least-mean-squares fit of the model to the data. To test our a priori predictions, we extracted contrast estimates from ROIs. These were spheres (r = 5 mm) centered on the peak coordinates discussed in the Introduction (X, Y, Z: right DLPFC: 32, 38, 26, Anderson et al., 2004; left mid-VLPFC: −50, 25, 14, Badre and Wagner, 2007; left cPFC: −52, 9, 24, Wimber et al., 2008). For the HC, we used the anatomical mask of the WFU pickatlas (Maldjian et al., 2003).

#### Effective Connectivity Analyses

To test the putative retrieval inhibition network supporting direct suppression, we modeled the effective connectivity between DLPFC and HC using DCM10. DCM explains regional effects in terms of dynamically changing patterns of connectivity during experimentally induced contextual changes (Friston et al., 2003). Importantly, this method allows inferences about the direction of causal connections, i.e., whether suppress events modulate the “top-down” connection from DLPFC to HC versus the reverse “bottom-up” connection. Therefore, we defined a standard model including both regions as nodes with bidirectional, intrinsic connections and within-region inhibitory autoconnections. This model was then modified to yield four model families that varied in the connections that could be modulated during suppress events (for details, see Results and Figure 3A). The driving input was modeled as a series of delta functions at any reminder onsets (i.e., for both suppress and recall events). Suppression was included as the modulatory input, defined as a change induced during the first second after the onset of suppress events.

The models were estimated separately for each session of each participant. We therefore extracted the regional time series of the BOLD signal for each participant of the direct suppression group (see Supplemental Experimental Procedures). Model fitting was based on these data and was achieved by adjusting the model parameters to maximize the free-energy estimate of the model evidence (Friston et al., 2003). BMS was then used to identify the family that could account best for the data (Penny et al., 2010). A random-effects approach was taken, since it does not assume that the optimal model will be the best for each individual (Stephan et al., 2010). This analysis reports the exceedance probability, i.e., the probability to which a given model is more likely than any other included model to have generated the data from a randomly selected participant.

We also conducted a PPI analysis (Friston et al., 1997) to test the hypothesized relationship between left cPFC-mid-VLPFC coupling and degree of memory competition. The physiological variable, i.e., the activation time series of cPFC, was obtained in an analogous way as for DCM. The psychological variable was defined as the contrast vector representing the task effect (suppress > recall). These regressors and their interaction term (i.e., the PPI regressor) were estimated at the first level. Contrast images associated with the PPI regressor were then entered into the regression analyses at the second level. SPMs were thresholded at p < 0.05, small-volume FWE corrected for the mid-VLPFC ROI.

## Supplemental Information

Document S1. Figures S1 and S2, Tables S1–S4, and Supplemental Experimental Procedures
