# Supplementary material for: Opposing Mechanisms Support the Voluntary Forgetting of Unwanted Memories
Source: Neuron. 2012 Oct 18;76-248(2):450–60. doi: 10.1016/j.neuron.2012.07.025 (PMC3480638; doi:10.1016/j.neuron.2012.07.025)
Supplement: Document S1. Figures S1 and S2, Tables S1–S4, and Supplemental Experimental Procedures [file mmc1.pdf]

Neuron, Volume *76*

Supplemental Information

Opposing Mechanisms Support the Voluntary Forgetting of Unwanted Memories

Roland G. Benoit and Michael C. Anderson

Supplemental Text

Supplemental behavioral results

As reported, an ANOVA on the recall data from the Same Probe test yielded a significant effect of retrieval status (suppress, recall, baseline) that did not interact with group. This effect partly reflected below-baseline forgetting of the suppressed items. We conducted two further follow-up ANOVAs with group as between-subject factor and retrieval status as within-subject factor (either baseline vs. recall or recall vs. suppress) to test whether repeated retrieval in the recall condition facilitated memory performance (Roediger and Butler, 2011). Only the effect of recall vs. suppress was significant, indicating that participants remembered less items that were previously suppressed than those which they had repeatedly recalled ($F_{(1,34)} = 26.87$, $p < 0.001$). Thus, though participants recalled more memories that were repeatedly recalled than suppressed, there was no significant benefit for the recall compared with the baseline items.

Though the initial ANOVA on the Independent Probe data did not show an overall effect of retrieval status (suppress, recall, baseline), the critical a priori analysis of suppress vs. baseline items revealed significant forgetting. Importantly, as reported, this inhibition effect did not interact with group, showing that the pattern of cue-independent forgetting was quite similar regardless of strategy. Despite the null effect of the overall ANOVA, we conducted further exploratory post hoc analyses to test the other retrieval-status differences (i.e., baseline vs. recall, and recall vs. suppress), and their interaction with group (thought substitution, direct suppression). The corresponding ANOVAs indicated that recall items were remembered worse than were baseline items ($F_{(1,34)} = 4.37$, $p < 0.05$), while the difference between recall and suppress items was not significant ($F_{(1,34)} = 0.21$, $p = 0.649$). Thus, whereas both tests yielded below-baseline forgetting of suppress items, the relative memory performance for recall items appeared to differ between the two tests. Indeed, this observation is consistent with a recent clear demonstration that recall performance on the IP test can at times be diminished for repeatedly recalled items due to

encoding specificity effects (Paz-Alonso et al., 2009; see also Thomson and Tulving, 1970; Murphy and Wallace, 1974). More generally these findings are consistent with prior data from 1300 individuals (Anderson and Huddleston, 2011; Levy and Anderson, 2008) demonstrating that facilitation effects are absent on independent probe tests.

Left hippocampus region-of-interest analysis

As shown in Figure S1B, the pattern of contrast estimates derived from the left hippocampus resembled the one from the right hippocampus. This was corroborated by the statistical analyses. The direct suppression group exhibited significantly lower activation during suppress than during recall trials ($t_{(17)} = 4.86$; $p < 0.001$), whereas this was not the case for the thought substitution group ($t_{(17)} = 0.473$; $p = 0.642$). Moreover, as for the right hippocampus, the activation difference for the suppress versus recall conditions differed between the two groups ($t_{(34)} = -2.07$, $p < 0.05$). We finally conducted an ANOVA on the contrast estimates of suppress versus recall with the factors hemisphere (right, left) and group (direct suppression, thought substitution), to examine whether the relative engagement for the two groups differed between the two hemispheres. As expected, only the group factor was significant ($F_{(1,34)} = 4.27$, $p < 0.05$), indicating that the direct suppression group showed a greater effect across the left and right hippocampi (for both the hemisphere effect and the interaction $F < 1$).

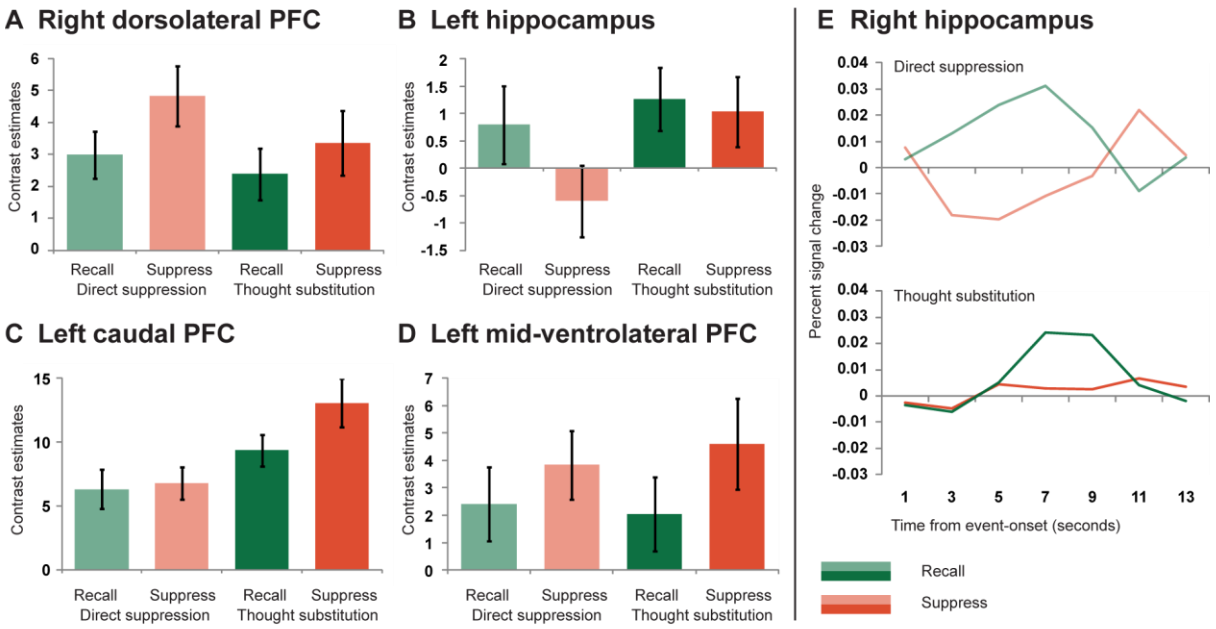

Figure S1, related to Figure 2: Region-of-interest contrast estimates per condition and group: (A) right dorsolateral prefrontal cortex, (B) left hippocampus, (C) left caudal prefrontal cortex, (D) left mid-ventrolateral prefrontal cortex, and (E) timecourses from the right hippocampus. Data are represented as mean +/− SEM.

Exploratory whole-brain analyses

For whole-brain analyses, contrast estimates for suppress and recall events were entered into a repeated-measures ANOVA using non-sphericity correction, with individuals and group (thought substitution, direct suppression) as between-subject factors. For exploratory purposes, the SPMs were thresholded at $p < 0.001$, uncorrected, and at least 5 contiguous voxels.

Table S1, related to Figure 2: Regions in which BOLD signal was significantly greater during suppress than recall trials in the direct suppression group.

			MNI (peak)				
**Region**	**~BA**	**Hemi.**	**x**	**y**	**z**	**Voxels**	***Z* max**
SFG	11	r	36	50	19	10	3.42
SFG, MiFG	9	r	36	38	31	5[a]	3.26
IFG	47	r	39	29	-5	251	4.22
			33	29	4	Same cluster	
			21	8	7	Same cluster	
ACC	32	r	9	29	28	20	4.02
MiFG	46	l	-33	26	22	11	3.61
IFG	47	l	-45	17	1	57	4.07
			-36	20	-5	Same cluster	
SFG	6/8	r/l	9	8	58	243	4.64
			15	14	67	Same cluster	
			6	17	55	Same cluster	
dorsal striatum		l	-24	8	4	19	3.85
PCG	6/9	l	-45	2	37	68	4.49
			-33	8	37	Same cluster	
MiFG, IFG	6/9	r	27	-7	52	257	5.20
			42	-1	40	Same cluster	
			54	5	34	Same cluster	
precuneus, IPL	7/40	r	21	-52	52	84	4.18
			30	-49	52	Same cluster	
			36	-40	46	Same cluster	
precuneus	7	l	-27	-52	52	11	3.28
MOG	19	r	51	-79	-8	11	3.56

ACC: anterior cingulate cortex; IFG: inferior frontal gyrus; IPL: inferior parietal lobule; MiFG: middle frontal gyrus; MOG: middle occipital gyrus; PCG: precentral gyrus; SFG: superior frontal gyrus; Hemi: hemisphere; l: left; r: right; thresholded at $p < 0.001$ and at least 5 contiguous voxels; [a] includes DLPFC.

Table S2, related to Figure 2: Regions in which BOLD signal was significantly greater during recall than suppress trials in the direct suppression group.

			MNI (peak)				
**Region**	**~BA**	**Hemi**	**x**	**y**	**z**	**Voxels**	***Z* max**
MeFG	10	l/r	-6	65	10	47	4.22
			12	53	13	Same cluster	
			6	59	16	Same cluster	
MeFG	11	r/l	6	50	-11	450	4.72
			-3	56	-11	Same cluster	
			6	32	-17	Same cluster	
MiFG	11	r	27	41	-5	8	3.34
ACC	24	r	9	29	10	10	3.91
BG		l	-6	23	10	16	3.96
SG	25	l/r	-6	8	-11	49	4.10
PHG, AMY		r	24	2	-23	53	4.59
ACC	24	r	6	-1	31	19	3.84
PHG, AMY		l	-27	-1	-20	19	3.67
insula	13	l	-36	-13	4	56	4.52
		l	-39	-19	16	Same cluster	
insula	13	r	36	-13	7	81	4.10
			54	-13	19	Same cluster	
			51	-7	28	Same cluster	
ITG, MTG	20/21	r	66	-19	-23	135	4.73
			60	-34	-23	Same cluster	
			63	-19	-11	Same cluster	
STG	41		54	-22	4	11	3.58
PHG	28	l	-21	-25	-8	22	4.13
STG	42	r	60	-28	16	53	3.55
			51	-28	19	Same cluster	
PHG, HC	36/35	r	36	-31	-14	62	4.32
			27	-22	-20	Same cluster	
PHG, HC	27	r	27	-31	-2	10	3.43
PoCG	3	r	18	-40	64	9	3.29
MTG	21	r	63	-43	-5	46	4.60
MTG, STG	21/42	l	-66	-49	-8	374	4.18
			-48	-37	25	Same cluster	
			-54	-31	16	Same cluster	
MTG	39	l	-54	-61	7	8	3.36
precuneus, MTG	39	r	39	-67	37	288	5.23
			39	-70	16	Same cluster	
			51	-61	22	Same cluster	
precuneus	19	l	-36	-76	37	197	4.61
cuneus, precuneus, PCC	18/30/31	r	21	-97	7	4346	6.23
			12	-67	25	Same cluster	
			21	-61	13	Same cluster	

ACC: anterior cingulate cortex; AMY: amygdala; BG: basal ganglia; HC: hippocampus; ITG: inferior temporal gyrus; MeFG: medial frontal gyrus; MiFG: middle frontal gyrus; MTG: middle temporal gyrus; PCC: posterior cingulate cortex; PHG: parahippocampal gyrus; PoCG: posterior central gyrus; SG: subcallosal gyrus; STG: superior temporal gyrus; Hemi: hemisphere; l: left; r: right; thresholded at $p < 0.001$ and at least 5 contiguous voxels.

Table S3, related to Figure 2: Regions in which BOLD signal was significantly greater during suppress than recall trials in the thought substitution group.

			MNI (peak)				
**Region**	**~BA**	**Hemi**	**x**	**Y**	**Z**	**Voxels**	***Z* max**
IFG, insula	47 / 13	r	30	26	-2	420	5.35
			15	5	10	Same cluster	
			51	17	-11	Same cluster	
MiFG	46	r	39	26	28	12	3.51
IFG	45	r	57	17	10	5	3.23
IFG, MiFG, MeFG	9/13/32	l/r	-51	8	31	1734 [a]	5.42
			-39	26	10	Same cluster [b]	
			-6	8	52	Same cluster	
MiFG, IFG	6/9	r	39	5	46	183	4.02
			48	2	34	Same cluster	
			24	2	43	Same cluster	
midbrain		l/r	0	-13	-20	18	4.57
STG, MTG	41/22	r	42	-37	4	22	3.74
			48	-31	-2	Same cluster	
IPL	40	r	36	-49	46	157	4.82
FuG	37	l	-42	-49	-14	6	3.37
cerebellum		r	30	-58	-29	6	3.49
IPL, SPL	40/7	l	-36	-61	46	657	5.28
			-30	-49	40	Same cluster	
			-24	-70	46	Same cluster	
cerebellum	18	r	12	-76	-26	17	3.61

FuG: fusiform gyrus; IFG: inferior frontal gyrus; IPL: inferior parietal lobule; MeFG: medial frontal gyrus; MiFG: middle frontal gyrus; MTG: middle temporal gyrus; SPL: superior parietal lobule; STG: superior temporal gyrus; Hemi: hemisphere; l: left; r: right; thresholded at $p < 0.001$ and at least 5 contiguous voxels.
[a] includes cPFC; [b] includes mid-VLPFC.

Table S4, related to Figure 2: Regions in which BOLD signal was significantly greater during recall than suppress trials in the thought substitution group.

			MNI (peak)				
Region	~BA	Hemi	x	y	Z	Voxels	*Z* max
MeFG	10	l/r	3	62	16	660	4.57
			-9	53	4	Same cluster	
			9	56	13	Same cluster	
MeFG	10	r	21	44	-2	5	3.42
SFG	9	l	-18	35	40	6	3.23
MeFG, SFG, MiFG	6/9	r	21	29	37	43	4.67
MTG, ITG	21/20	l	-63	-13	-17	61	4.21
			-60	2	-23	Same cluster	
			-48	-7	-23	Same cluster	
insula	13	l	-36	-16	4	58	4.69
ITG	20	r	60	-19	-23	6	3.29
insula	13	r	48	-25	25	67	4.16
			39	-19	25	Same cluster	
PCG	2/5	l	-57	-28	19	46	3.76
PCG	2/5	r	24	-40	67	17	3.85
PCG/SPL	5/7	l	-18	-46	64	24	3.98
MTG	21/22	r	66	-49	-2	28	4.12
			12	-52	7	Same cluster	
MTG	21	l	-66	-52	-5	6	3.51
PCC	23/31/29	r/l	0	-58	19	64	3.75
			18	-61	16	Same cluster	
AG	39	r	48	-70	28	16	3.76
LG, MOG	18/19	l	-27	-73	-5	12	3.82
AG	39	l	-45	-79	31	31	3.57
precuneus	19	r	18	-85	37	7	3.23
LG, precuneus	17/18/19	r	12	-94	-5	270	5.08
			24	-76	-5	Same cluster	
			21	-91	-5	Same cluster	

AG: angular gyrus; ITG: inferior temporal gyrus; LG: lingual gyrus; MeFG: medial frontal gyrus; MiFG: middle frontal gyrus; MOG: middle occipital gyrus; MTG: middle temporal gyrus; PCC: posterior cingulate gyrus; PCG: postcentral gyrus; SFG: superior frontal gyrus; SPL: superior parietal lobule; Hemi: hemisphere; l: left; r: right; thresholded at $p < 0.001$ and at least 5 contiguous voxels.

Dynamic causal modelling

Partitioning the model space in a congruent versus incongruent family

As reported in the manuscript, Bayesian Model Selection (BMS) indicated that family IV comprised the superior models. Thus, a family could account best for the data that was consistent with the hypothesized "top-down" influence from DLPFC to HC. The same picture emerged when we first grouped the models into two meta-families that were either inconsistent (no "top-down" modulation) versus consistent (includes "top-down" modulation) with the hypothesized inhibitory mechanism (family exceedance probabilities: inconsistent models: 0.12; consistent models: 0.88) (Fig. S2a). This analysis was followed-up by BMS between the two model families nested within the winning family (i.e., family III and IV). Consistent with the initial result, this BMS favored family IV (exceedance probability: 0.84) over family III (exceedance probability: 0.16) (Fig. S2b).

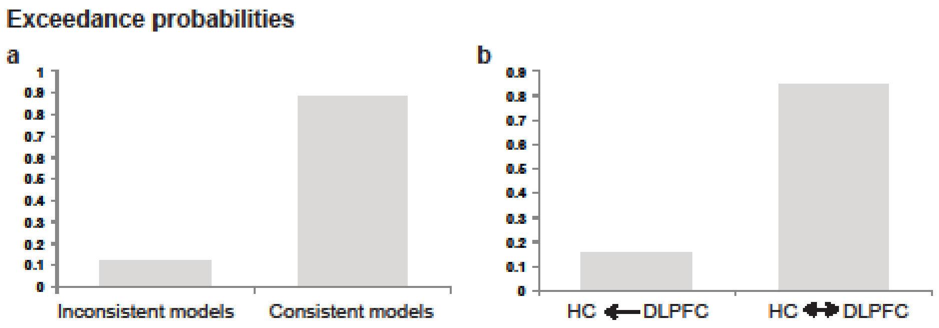

Figure S2, related to Figure 3: Bayesian Model Selection. (a) Meta model families inconsistent versus consistent with the hypothesized effect of suppression on "top-down" dorsolateral prefrontal cortex - hippocampal connectivity. (b) Models from the winning, consistent, family.

Bayesian model selection: fixed-effects approach

For model selection, we adopted a random-effects approach, because it does not assume that the optimal model will be the best for each individual (Stephan et al., 2010). However, here we assessed whether the same family would also be selected with a fixed-effects approach that makes this assumption. Again, family IV turned out to be the superior model with a family posterior probability of ~1, i.e., very strong evidence that this model family generated the observed group data.

Supplemental Experimental Procedures

DCM ROI selection

The models were estimated separately for each session of each participant. We therefore extracted the regional time-series of the BOLD signal for each participant of the direct suppression group. First, we identified the group peaks within a 10 mm sphere centered on our DLPFC ROI (Anderson et al., 2004) (suppress > recall), and within the right HC mask of the WFU pickatlas (Maldjian et al., 2003) (suppress < recall). (Note that our analyses were restricted to the right HC, since only this region exhibited univariate effects for the contrast [suppress > recall] that survived small-volume FWE correction.) For each participant, we then identified the individual peak voxel within a 10 mm sphere centered on the observed group peaks (XYZ: DLPFC: 36 38 31; HC: 33 -28 -14). For the HC, the individual peaks also had to be within the HC mask. By considering subject-specific peaks, we ensure that our connectivity analyses are based on those voxels that are most strongly engaged for each individual (Stephan et al., 2010). That is, otherwise the DCM parameter estimation would potentially be more reliable for those subjects whose peak activity within DLPFC was closest to the group peak (in so far as individual peaks can be taken to indicate the neuronal population that is most strongly engaged for the given task in a specific subject). This, in turn, would potentially have biased the between-subject analysis of the DCM parameter estimates, i.e., the median split by inhibition. The individual peaks then served as subject-specific centers for spherical ROIs (radius: 5 mm). The first eigenvariate from a ROI (i.e., the first principal component of the time-series of the voxels), adjusted for the effects of interest, constituted the regional activation.

PPI ROI selection

ROI selection for the psychophysiological interaction analyses followed the identical procedure as for the DCM analyses. We first identified the thought substitution group peak of the task contrast (suppress > recall) within the a priori cPFC ROI (Wimber et al., 2008), before detecting the subject specific peaks that were closest to the group-level peak (XYZ: -51 8 31). These coordinates served as centers for spherical ROIs. The first eigenvariate from a ROI, adjusted for the effects of interest, constituted the physiological variable.

Supplemental References

Levy, B.J., and Anderson, M.C. (2008). Individual differences in the suppression of unwanted memories: The executive deficit hypothesis. Acta. Psychol. (Amst). *127,* 623-635.

Murphy, M.D., and Wallace, W.P. (1974). Encoding specificity: semantic change between storage and retrieval cues. J. Exp. Psychol. *103*, 768–774.

Paz-Alonso, P.M., Ghetti, S., Matlen, B.J., Anderson, M.C., and Bunge, S.A. (2009). Memory suppression is an active process that improves over childhood. Front. Hum. Neurosci. *3*, 1-6.

Thomson, D.M., and Tulving, E. (1970). Associative encoding and retrieval: weak and strong cues. J. Exp. Psychol. *86*, 255–262.
